# When aposematism is not enough: Exotic *Rattus rattus* shows no mercy for carcasses of *Salamandra salamandra* in insular populations

**DOI:** 10.1002/ece3.11229

**Published:** 2024-05-15

**Authors:** Guillermo Velo‐Antón

**Affiliations:** ^1^ Facultad de Biología Universidad de Vigo Vigo Spain

**Keywords:** aposematic, behavioural shift, camera traps, predator–prey interactions

## Abstract

Predator–prey interaction is a major force driving natural selection. Yet, the identification of species preying on, or consuming, aposematic species is largely unknown. Here, I conduct a study evaluating the role of the exotic *Rattus rattus* as a consumer and possible predator of the aposematic and toxic *Salamandra salamandra*. I used camera traps to investigate the response of *R. rattus* towards *S. salamandra* carcasses in two insular populations, Ons and San Martiño (NW Spain), which show remarkable contrasting behaviour (nocturnal vs. diurnal activity) and demographic and phenotypic differences. This study unveils *R. rattus* consumes *S. salamandra* despite its aposematic colour pattern and toxicity. The high number of salamander carcasses consumed or taken by rats throughout each island (90%–100%) and the lack of other possible predator–prey interactions points to *R. rattus* as an efficient consumer of *S. salamandra* in these insular environments, which might exert a high predation pressure on both islands. Yet, the drivers underlying the behavioural and phenotypic differences in these insular populations should be further investigated.

## Graphical abstract

Predator–prey interaction is a major force driving natural selection. *Rattus rattus* consume aposematic and toxic *Salamandra salamandra* in two insular populations (NW Spain). *Rattus rattus* seems to exert a high predation pressure on *S. salamandra*, particularly on the small and diurnal population of San Martiño island.

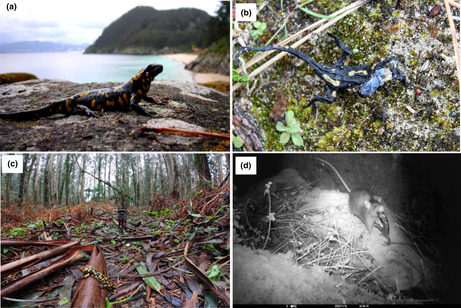

## 1 INTRODUCTION

Aposematism is commonly referred to as the advertisement by a species—showing conspicuous coloration and defences—to a potential predator and is thus considered an anti‐predator strategy (Barzaghi et al., 2022; Caro & Ruxton, 2019; Skelhorn et al., 2016). Aposematic species can rely on primary (i.e., striking colours and contrasting patterns) and secondary (e.g., weaponry, malodor and toxins) defences. The combination of yellow/red and black coloration is a reliable primary defence in aposematic species (e.g., Rojas et al., 2021), because predators recognise it as an alarm signal and associate it with bad experiences (e.g., toxicity and unpalatability) from previous attacks.

The fire salamander, *Salamandra salamandra*, is a renowned aposematic amphibian that relies on their contrasting black and yellow coloration pattern (although other colours such as red can also be present; Velo‐Antón & Buckley, 2015) and the toxins (alkaloids) secreted from skin glands to alert possible predators of their toxicity and unpalatability (Lüddecke et al., 2018). Yet, some species were found to sporadically consume adults of fire salamanders, such as birds of prey (e.g., *Buteo buteo*; Bustamante, 1985) and mammals (*Sus scrofa*; Carretero & Rosell, 1999; *Lutra lutra*, Morales et al., 2004). However, these studies relied on indirect evidence (i.e., gut contents, faecal DNA metabarcoding or clay models), and thus, they did not provide direct evidence of either fire salamander consumption or their role as predators or scavengers of this aposematic species.

**FIGURE 1:**
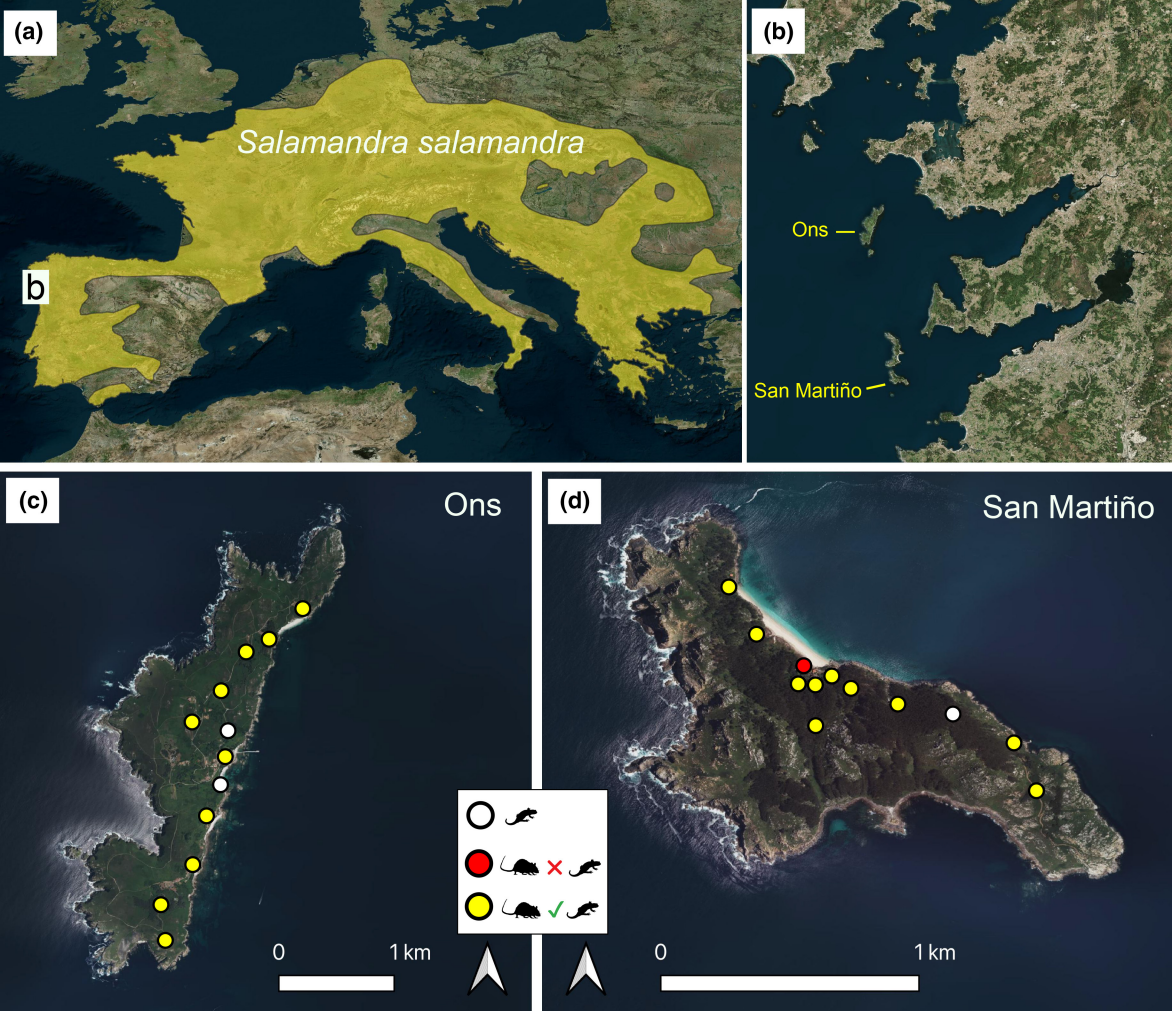
(a) Map showing the range of *Salamandra salamandra* across Europe. The inset represents the location of the study area in NW Spain; (b) location of the two studied insular populations (Ons and San Martiño) and neighbouring main islands; (c) distribution of camera traps in Ons; (d) distribution of camera traps in San Martiño. The figure legend indicates those camera trap with (i) no records of *Rattus rattus* (white circles); (ii) presence of *R. rattus* but no signs of scavenging by rats on *S. salamandra* carcasses (red circles) and (iii) records confirming *R. rattus* consuming or taking away the carcasses.

An exceptional behavioural shift has been observed in one of the very few insular populations of *S. salamandra* during a long‐term monitoring since 2003 (Velo‐Antón & Cordero‐Rivera, 2017). In San Martiño, an off‐shore island located in NW Spain, a population of fire salamanders shows diurnal activity, avoiding the more beneficial nocturnal behaviour typical of this species (Velo‐Antón & Buckley, 2015). Contrarily, the neighbouring island of Ons (12 km apart; Figure 1) harbours a nocturnal *S. salamandra* population. Both populations got isolated from the mainland with the formation of the islands when sea level arose ca. 8000 years ago, and independently evolved to pueriparity (a type of viviparity where females give birth to fully developed juveniles; Velo‐Antón et al., 2015), which makes terrestrial predators the only possible consumers of insular salamanders. These two insular populations also differ in: (i) genetic diversity (lower in San Martiño; Velo‐Antón et al., 2012); (ii) body size and condition, being smaller and slimmer in the diurnal population of San Martiño (Velo‐Antón & Cordero‐Rivera, 2017); and (iii) population size, being larger in Ons (Velo‐Antón et al., 2012).

A different response to the predation pressure of the black rat, *Rattus rattus,* on *S. salamandra* was suggested as the driving force underlying the behavioural shift in San Martiño through natural selection. This was hypothesised based on the carcasses of fire salamanders found half eaten on this island (Velo‐Antón & Cordero‐Rivera, 2011), and a posterior experimental study using plasticine models and camera traps (Velo‐Antón & Cordero‐Rivera, 2017), which pointed to a selective pressure of exotic rats over the small salamander population in San Martiño. However, no direct evidence of *R. rattus* consuming aposematic fire salamanders has been reported to date, and the role of rats as possible predators of fire salamanders has only been suspected (Pezaro et al., 2018; Velo‐Antón & Cordero‐Rivera, 2017). Here, I take advantage of the use of carcasses and camera trapping (a method with recent interest studying scavenging behaviours, e.g., Redondo‐Gómez et al., 2023) to show, for the first time, *R. rattus* as a consumer of the aposematic and toxic *S. salamandra*, while speculate about its role as a predator of these phenotypic and behavioural divergent insular salamander populations.

## 2 METHODS

In November 2023, I used 12 camera traps on each island (Figure 1) to monitor the carcasses of *S. salamandra* for 48 h (less than a week interval between experiments). San Martiño (146 ha; altitude: 0–175 m a.s.l.) is separated by 3.6 km from the mainland, and uninhabited (only sporadically visited by researchers and by tourists during the summer). Ons (428 ha; altitude: 0–128 m a.s.l.) is separated by 6 km from the mainland, and continuously inhabited by a few locals and staff from the National Park (also visited by tourists in summer). Fresh carcasses of *S. salamandra* are easily found due to roadkill events and, more rarely, floods during heavy rainy nights. In late October and early November 2023, during regular nocturnal monitoring of *S. salamandra* in nearby continental populations, I found dozens of death salamanders, presumably deceased in the same nights, and collected 24 that showed no clear external injuries and maintained the body shape, and kept them at −20°C until the day before their deployment, when they were defrosted at room temperature. On each island, I deployed the carcasses in the morning and left them only for 48 h because I noticed from previous trials that the body shape and especially the skin rapidly deteriorated after this period. I used infrared motion‐triggered cameras (Apeman H40) to record possible *S. salamandra* predators or scavengers and their behaviours using 20‐ to 30‐s videos (taken after automatic detection of movement; interval between consecutive videos: 5 s) and placed one carcass at 25–40 cm from each camera. I distributed the cameras throughout each island in the most humid places, near the streams and water ponds, when possible (Figure 1), coinciding with the home range observed for *S. salamandra* and avoiding the western areas that are exposed to strong winds from the Atlantic and where *S. salamandra* has been rarely found (Velo‐Antón & Cordero‐Rivera, 2017). I checked every carcass after 24 h, removing those cameras that had no carcass left and removed the remaining cameras after 48 h of the beginning of the experiment. I checked all the videos and recorded, for each island, the number of cameras where *R. rattus* was detected and the number of consuming events (i.e., when carcasses were taken or consumed) by rats on *S. salamandra* carcasses, as well as the presence of other possible *S. salamandra* predators.

## 3 RESULTS

**FIGURE 2:**
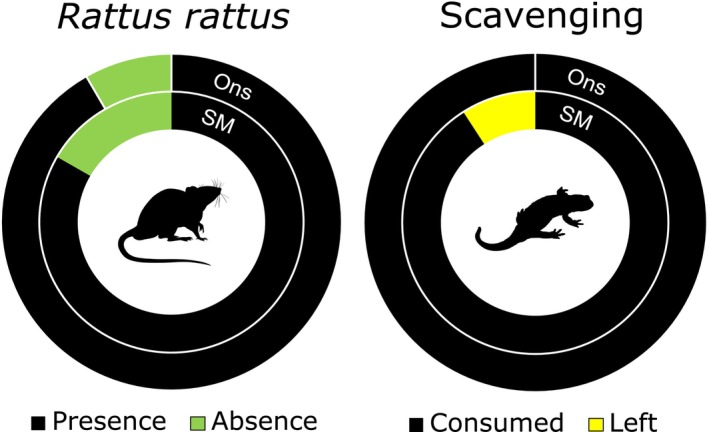
Schematic figure summarising the presence (%) of *Rattus rattus* recorded in the cameras in each island, and the observed scavenging behaviour (%) of *R. rattus* on *Salamandra salamandra* carcasses in each island. San Martiño (SM) and Ons are represented in the inner and outer circle, respectively.

The videos recorded in this study show both the presence of *R. rattus* and the consumption of *S. salamandra* in most of the cameras deployed for 2 days in the islands. The presence of *R. rattus* was recorded throughout each island in 11 (92%) and 10 (83%) cameras deployed in San Martiño and Ons, respectively (Figures 1 and 2). From those cameras, a total of 10 events were recorded in each island where *R. rattus* took, or consumed, *S. salamandra* carcasses (90% of the carcasses with recorded presence of rats in San Martiño; 100% in Ons; Figure 2). All these events occurred at night and mostly during the first night (60% in San Martiño; 90% in Ons). The average number of consuming events per camera was 0.9 and 0.8 in San Martiño and Ons, respectively, while the percentage of rats consuming carcasses was 66% and 53% in San Martiño and Ons, respectively.

**FIGURE 3:**
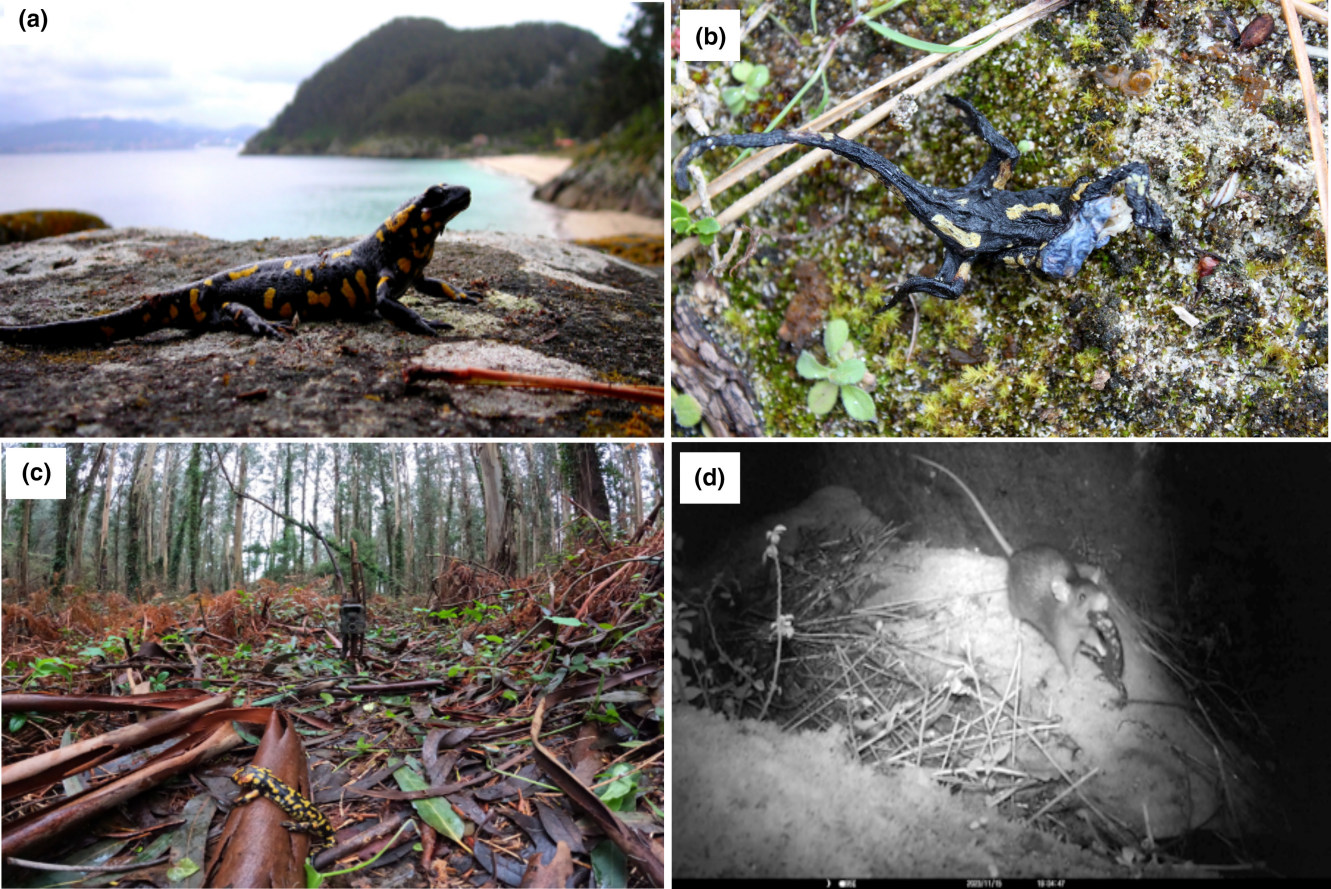
(a) Diurnal *Salamandra salamandra* in San Martiño; (b) *S. salamandra* carcass half eaten found in San Martiño island; (c) camera trap deployed in San Martiño with a carcass of *S. salamandra*; (d) camera trap image of *Rattus rattus* holding a *S. salamandra* carcass.

Some of the videos obtained were of low quality because the weather conditions fogged up the lens of some cameras, and thus representative videos of the behaviour of rats—when salamander carcasses were present—are summarised in a single video per island (San Martiño, Video 1, https://youtu.be/ObxTu_YwV3M; Ons, Video 2, https://youtu.be/5wOqE3KFG0I). In San Martiño, rats generally approached the carcasses and grabbed salamanders from their head or neck (Figure 3d), then started chewing and manipulating the carcass before they took them away. Also, some of the rats quickly approached and took away the carcasses, while one rat smelled the carcass but showed no apparent consumption. In Ons, the rats approached and chewed the carcasses, but in some cases, the rats took longer to interact with them, and some were taken by the rats from the tail or hindlimbs. Two fear‐related events were detected in Ons, with rats hesitating to get closer to the carcasses, moving and stopping for some seconds, until they bit and chewed the carcasses. In San Martiño, *Neovison vison* was recorded once in one camera but showing no consumption when passed next to the carcass (https://www.youtube.com/watch?v=9gPI6b7LrvY). Some fear‐related—hesitancy and flight response—events were recorded for *Apodemus sylvaticus* in Ons, and although one individual finally approached a carcass and seemed to chew it for a few seconds, it then moved away leaving the carcass in the exact same position. No other possible scavengers were recorded by any of the cameras on both islands.

**VIDEO 1:** Frame of a series of videos recorded by camera traps deployed on San Martiño island (NW Spain) showing a black rat, *Rattus rattus*, grabbing a fire salamader, *Salamandra salamandra*, carcass from the neck.

**VIDEO 2:** Frame of a series of videos recorded by camera traps deployed on Ons island (NW Spain) showing a black rat, *Rattus rattus*, chewing a fire salamader, *Salamandra salamandra,* carcass.

## 4 DISCUSSION

*Rattus rattus* is considered an omnivorous vertebrate, including a variety of plant and invertebrate materials (Clark, 1982), but also as a occasional predator or scavenger of birds in insular environments (Major, 1991; Muletz‐Wolz et al., 2021). This study shows novel evidence of *R. rattus* consuming aposematic *S. salamandra* carcasses, despite its chemical defence. The high levels of fire salamander consumption (90%–100%) by rats observed in these insular environments suggest the aposematic pattern of this species might not be an effective anti‐predatory strategy when facing the exotic *R. rattus*. It also shows a direct evidence of *S. salamandra* consumption, which has been previously reported from indirect evidence (e.g., stomach content, clay models, DNA metabarcoding, stable isotope analysis) obtained from presumed predators.

However, whether fire salamanders are scavenged or predated remains understudied. Obtaining direct evidence to evaluate the role of *R. rattus* as a predator of *S. salamandra* will require a different methodological approach, which would have to be innovative since *S. salamandra* is considered Vulnerable by the IUCN and thus using live salamanders as bait in such experiments is not allowed. On the other hand, a proper evaluation of *R. rattus* as an efficient scavenger on *S. salamandra* should also consider different states of carcass degradation. This short‐term experiment—2 days—and the rapid consumption of most of the carcasses deployed on these islands did not allow a severe degradation of the carcasses, and thus, the role of *R. rattus* as scavenger is still not well‐understood and would require a different methodological setup (e.g., using carcasses in different states of decomposition). Yet, this study exemplifies the value of combining camera trapping and carcasses to further explore this complex and elusive topic.

Since no other possible predators were recorded consuming the carcasses in any of the islands and plasticine salamander models were only bitten by rats (Velo‐Antón & Cordero‐Rivera, 2017), it is reasonable to suggest *R. rattus* constitutes the main, perhaps the only, predator of insular *S. salamandra*. This is also supported by the broad distribution of *R. rattus* in both islands, the high number of carcasses taken in a very short time span (1–2 nights), and the appearance of *S. salamandra* carcases partially (head and forelimbs) consumed and observed in previous samplings (Velo‐Antón & Cordero‐Rivera, 2011), which coincides with the feeding behaviour of most recorded rats. Another possible predator of fire salamanders, the invasive American mink, *N. vison*, was also recorded during the experiment but does not seem to have an impact on these insular salamanders because of its occasional presence on any of these islands, the lack of interaction with fire salamander carcasses (also supported by personal observations on a nearby island) and plasticine models, and the absence of salamander's bone and skin within the digestive tracts of some dissected minks (Velo‐Antón & Cordero‐Rivera, 2011).

The high consumption of salamander carcasses by rats observed in this study suggests rats as a possible cause of the recent extinctions of *S. salamandra* in the nearby islands of Faro and Monteagudo during the late 1990s (Galán, 2003), which together with San Martiño comprise the Cíes archipelago and where *R. rattus* is largely present. *Rattus rattus* and *S. salamandra* have been likely co‐evolving in a relatively short evolutionary time scale, since the rats were accidentally introduced by sailors in medieval times (Yu et al., 2022). For the present populations, although a higher predation pressure exerted by the abundant *R. rattus* on the small salamander population in San Martiño might trigger the behavioural shift—nocturnal to diurnal behaviour—this cannot be supported with the data at hand because the proportion of attacked carcasses was similar between islands. Thus, the drivers underlying the behavioural and phenotypic differences observed between insular salamander populations are not yet well understood and would require further investigation. Community‐level approaches and behavioural experiments (e.g., Valeix et al., 2009) targeting both the predator behaviour and the prey response to predator cues (e.g., chemical, Ibáñez et al., 2014) are thus needed to further investigate the hypothesis of the behavioural shift mediated by a predation‐risk strategy, as well as to understand how this could alter the food‐web connectivity of this relatively small island.

## AUTHOR CONTRIBUTIONS

**Guillermo Velo‐Antón:** Conceptualization (lead); data curation (lead); formal analysis (lead); funding acquisition (lead); investigation (lead); methodology (lead); project administration (lead); resources (lead); visualization (lead); writing – original draft (lead); writing – review and editing (lead).

## CONFLICT OF INTEREST STATEMENT

The author declares no competing interests.

## Data Availability

A selection of two videos summarising the predator–prey interactions recorded from both islands are embedded in the manuscript. Untrimmed videos obtained from camera traps are holded in Figshare: https://doi.org/10.6084/m9.figshare.24802113.
